# Effects of carbohydrate supplementation on aerobic exercise performance during acute high altitude exposure and after 22 days of acclimatization and energy deficit

**DOI:** 10.1186/s12970-020-0335-2

**Published:** 2020-01-09

**Authors:** Karleigh E. Bradbury, Claire E. Berryman, Marques A. Wilson, Adam J. Luippold, Robert W. Kenefick, Andrew J. Young, Stefan M. Pasiakos

**Affiliations:** 1 Thermal and Mountain Medicine Division, US Army Research Institute of Environmental Medicine, 10 General Greene Avenue, Bldg, Natick, MA 42 USA; 2 Military Nutrition Division, US Army Research Institute of Environmental Medicine, 10 General Greene Avenue, Bldg, Natick, MA 42 USA; 3 Oak Ridge Institute for Science and Education, 4962 Millennium Drive, Suite, Belcamp, MD 101 USA

**Keywords:** Hypoxia, Time trial, Ergogenic aid

## Abstract

**Background:**

The ergogenic effects of supplemental carbohydrate on aerobic exercise performance at high altitude (HA) may be modulated by acclimatization status. Longitudinal evaluation of potential performance benefits of carbohydrate supplementation in the same volunteers before and after acclimatization to HA have not been reported.

**Purpose:**

This study examined how consuming carbohydrate affected 2-mile time trial performance in lowlanders at HA (4300 m) before and after acclimatization.

**Methods:**

Fourteen unacclimatized men performed 80 min of metabolically-matched (~ 1.7 L/min) treadmill walking at sea level (SL), after ~ 5 h of acute HA exposure, and after 22 days of HA acclimatization and concomitant 40% energy deficit (chronic HA). Before, and every 20 min during walking, participants consumed either carbohydrate (CHO, *n* = 8; 65.25 g fructose + 79.75 g glucose, 1.8 g carbohydrate/min) or flavor-matched placebo (PLA, *n* = 6) beverages. A self-paced 2-mile treadmill time trial was performed immediately after completing the 80-min walk.

**Results:**

There were no differences (*P* > 0.05) in time trial duration between CHO and PLA at SL, acute HA, or chronic HA. Time trial duration was longer (*P* < 0.05) at acute HA (mean ± SD; 27.3 ± 6.3 min) compared to chronic HA (23.6 ± 4.5 min) and SL (17.6 ± 3.6 min); however, time trial duration at chronic HA was still longer than SL (*P* < 0.05).

**Conclusion:**

These data suggest that carbohydrate supplementation does not enhance aerobic exercise performance in lowlanders acutely exposed or acclimatized to HA.

**Trial registration:**

NCT, NCT02731066, Registered March 292,016

## Background

Reduced arterial oxygen content degrades aerobic exercise performance in lowlanders initially exposed to high altitude (HA) [1]. After several weeks of HA exposure, acclimatization occurs, and aerobic exercise performance partially improves [1–3]. Another factor that may impact the performance degradation seen at altitude are changes in substrate oxidation [4], as endogenous carbohydrate oxidation during exercise may be higher with acute HA exposure compared to absolute VO_2_-matched exercise at sea level (SL) [5]. After acclimatization, muscle glucose uptake during exercise is also increased relative to SL [6]. Collectively, these data suggest that consuming supplemental carbohydrate during exercise at HA might be an effective strategy to attenuate performance decrements by sparing endogenous carbohydrate stores and meeting the apparent increase in carbohydrate requirements during exercise.

Carbohydrate supplementation during exercise at SL spares endogenous carbohydrate stores and delays the onset of fatigue [7, 8]. Effects of carbohydrate supplementation on exercise performance at HA are not well described. In unacclimatized men, Fulco et al. [9] demonstrated that carbohydrate supplementation during exercise enhanced time trial performance compared to placebo after 3 days of HA (4300 m) exposure concomitant with a 30% energy deficit (negative energy balance is largely unavoidable and commonly experienced by lowlanders sojourning at HA) [10]. However, carbohydrate supplementation had no further performance benefit after 10 days of acclimatization and negative energy balance. In a follow-up study, Fulco et al. [11] found no benefit of carbohydrate supplementation on time trial performance on the first and third day of HA exposure in previously HA acclimatized men in a state of energy balance. These discordant results from vastly different experimental designs (i.e., energy and acclimatization status of participants) suggest that the potential ergogenic effects of supplemental carbohydrate on exercise performance at HA may, in part, be modulated by acclimatization. To extend these findings [9, 11], we examined the effects of carbohydrate supplementation on aerobic exercise performance in unacclimatized men after 5 h of HA (4300 m) exposure and following 22 days of HA acclimatization and concomitant 40% energy deficit. Based on the studies by Fulco and colleagues [9, 11], we hypothesized that carbohydrate supplementation would enhance exercise performance during acute HA exposure, but not after HA acclimatization.

## Methods

### Experimental design

Data included in this Short Report were secondary analyses from a controlled feeding and exercise study that assessed the effects of high protein diets on body composition during sustained energy deficit at HA [12]. This study (clinical trials.gov: NCT02731066) was approved by the Institutional Review Board at the US Army Research Institute of Environmental Medicine (USARIEM, Natick, MA) and participants provided written informed consent. Characteristics of the 14 unacclimatized, young men included in this report were detailed by Young et al. [13].

The experimental design has been reported extensively [12–16]. In brief, the study was conducted over 43 consecutive days. During the first 21 days (SL), participants consumed a self-selected, weight-maintaining diet, maintained habitual exercise levels, and were free-living but visited the laboratory daily. On SL day 21, participants were flown from Boston, MA to Denver, CO where they were placed on supplemental oxygen until being driven to the summit of Pikes Peak, CO (4300 m) the following morning where they resided at the USARIEM Maher Memorial Altitude Laboratory for the next 22 days at HA. During HA, participants were under constant supervision, performed daily exercise, and consumed either standard protein (mean ± SD; 1.1 ± 0.2 g/kg/d) or high protein (2.1 ± 0.2 g/kg/d), carbohydrate-matched, energy deficient diets (40%; 30% by energy restriction and 10% by exercise). Fat was the primary manipulated macronutrient during the energy deficit, such that the standard protein group consumed 1.1 ± 0.2 g/kg/d fat, and the high protein group consumed 0.7 ± 0.1 g/kg/d fat [12]. The diet intervention resulted in a 7.9 ± 1.9 kg loss of total body mass [13].

### Exercise and 2-mile time trial

Participants were randomized to groups and were provided equal volumes of flavor-matched carbohydrate (CHO; 65.25 g fructose + 79.75 g glucose ingested at 1.8 g/min, *n* = 8, 3 standard and 5 high protein) and placebo (PLA; *n* = 6, 4 standard and 2 high protein) beverages during 80 min of metabolically-matched, steady-state treadmill walking at SL (day 7), 5 h after arriving at HA (acute HA), and after 22 days of acclimatization and energy deficit (chronic HA, day 42). As previously reported [13] treadmill speed, grade, absolute oxygen uptake (L/min), and metabolic rate were not different across study phases. All time trials were completed at the same time of day (between 1130 and 1230) and participants were fasted prior to the start of exercise. Participants were given a 5 min rest after completing the steady-state exercise before performing a self-paced, 2-mile treadmill time trial. Peripheral oxygen saturation (SpO_2_; finger pulse oximetry, Model 9560; Nonin, Plymouth, MN, USA) and heart rate were assessed at baseline and in half mile intervals, and Ratings of Perceived Exertion (RPE) [17] were recorded at baseline and immediately after completing the 2-mile time trial. Participants were familiarized to the test procedures (80 min steady-state and 2-mile time trial) on three occasions before completing the SL trial on day 7. The coefficient of variation of the three familiarization time trials was 5.06%, indicating that the test-retest variation in performance was small.

### Statistical analyses

As mentioned, this study was part of a larger investigation [12], powered to test the effects of dietary protein on body composition during altitude acclimatization and concomitant energy deficit. We did not expect dietary protein level to affect time trial performance at chronic HA (neither SL or acute HA were subject to the dietary protein intervention), which we confirmed using linear mixed models with dietary protein level, treatment (CHO, PLA), phase (SL, acute and chronic HA), exercise time point (for SpO_2_ and heart rate), and their interactions as fixed effects. Therefore, the effects of the dietary protein intervention are not presented in this report, and the data were re-analyzed using the same linear mixed-model without dietary protein in the model. Tukey’s HSD tests were used for multiple comparisons if significant main effects of interaction effects were observed. Freidman’s test was used to assess differences in RPE (baseline, end of the time trial) across study phases. Statistical significance was accepted at *P* < 0.05. Data were analyzed using SPSS (v.22.0; Chicago, IL, USA).

## Results

**Fig. 1:**
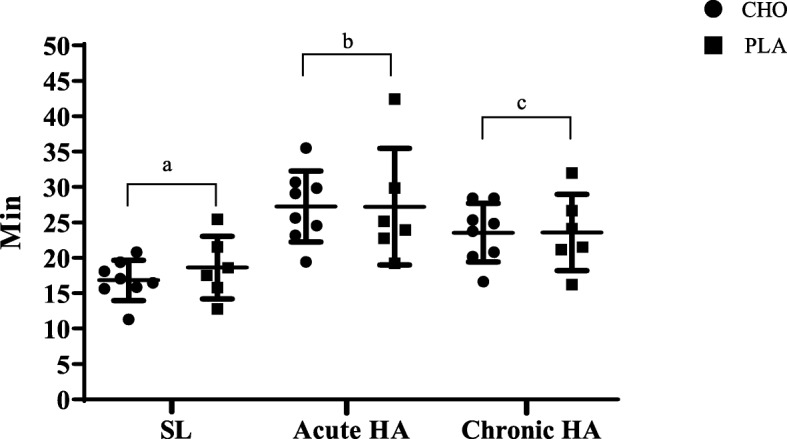
2-mile time trial duration (min) at sea level, acute and chronic HA for CHO and PLA. Values are presented as mean ± SD. Points not sharing the same letter are different (phase main effect, P < 0.05). SL, sea level; HA, high altitude; CHO, carbohydrate; PLA, placebo

There was no effect (*P* > 0.05) of CHO on time trial duration, oxygen saturation, heart rate, or RPE across study phases. Time trial duration was longer (*P* < 0.05) at acute HA (27.3 ± 6.3 min) compared to chronic HA (23.6 ± 4.5 min) and SL (17.6 ± 3.6 min); however, time trial duration at chronic HA was still longer than SL (phase main effect, *P* < 0.05, Fig. 1). Mean heart rate and SpO_2_ during the time trials were lower at acute and chronic HA compared to SL, and SpO_2_ at chronic HA was higher than acute HA (phase main effect, *P* < 0.05, Table 1). RPE was similar across study phases.

**Table 1:** Heart rate, peripheral oxygen saturation, and ratings of perceived exertion during the 2-mile time trial^a^

	SL		Acute HA		Chronic HA	
	CHO	PLA	CHO	PLA	CHO	PLA
HR (bpm)^2^	181 ± 10	175 ± 9	159 ± 9	168 ± 14	166 ± 13	161 ± 18
SpO_2_ (%)^2,3^	93 ± 2	95 ± 3	71 ± 4	73 ± 4	77 ± 3	81 ± 4
RPE	17 (14–19)	17 (16–18)	16 (13–20)	18 (15–20)	18 (14–19)	19 (16–19)

^a^Values presented as mean ± SD, except for RPE which is presented as median (range)

^2^Phase main effect; acute and chronic HA different than SL, *P* < 0.05

^3^Phase main effect; chronic HA different than acute HA, *P* < 0.05.

*SL* Sea level, *HA* High altitude, *HR* Heart rate, *CHO* Carbohydrate, *PLA* Placebo, *SpO*_*2*_ Peripheral oxygen saturation, *RPE* Rating of Perceived Exertion

## Discussion

This study examined the effects of consuming supplemental carbohydrate on aerobic exercise performance in recreationally-active, healthy, young lowlanders at HA, before and following 22 days of acclimatization while in a constant state of negative energy balance. We demonstrated that consuming supplemental carbohydrate during steady-state exercise had no performance-enhancing effects on time trial performance before or after HA acclimatization. As expected, time trial performance was approximately 55% slower at acute HA compared to SL. Acclimatization partially restored exercise performance relative to acute HA; however, time trial performance at chronic HA was still 34% slower than SL. Carbohydrate supplementation had no effect on perceived effort during any of the performance trials. These data, which were derived from a highly controlled exercise and diet intervention study, suggest that carbohydrate supplementation does not enhance aerobic exercise performance of lowlanders at HA.

In our study, supplemental carbohydrate did not enhance exercise performance in lowlanders acutely exposed to HA (5 h), which conflicts with data reported by Fulco et al. [9], who demonstrated that lowlanders consuming carbohydrate on the third day of continuous residence at 4300 m performed a 720 kJ cycle ergometry time trial faster than participants consuming the placebo. We suspect the discrepancy between our data and those by Fulco et al. [9] are due, in part, to the apparent inability to effectively oxidize exogenous carbohydrate upon initial exposure to HA. In the same study, we demonstrated a 52% reduction in exogenous carbohydrate oxidation and a corresponding increase in endogenous carbohydrate oxidation (i.e., blood glucose, muscle and liver glycogen) during the 80 min steady-state exercise bout with acute HA exposure compared to SL^13^. The mechanisms accounting for the reduction in exogenous carbohydrate oxidation during acute HA exposure are not known, but may be attributable to adjustments in glycemic regulation, as participants were markedly hyperinsulinemic, which inhibited lipolysis and likely accelerated glycogenic flux. The inability to effectively oxidize exogenous carbohydrate during acute HA exposure was alleviated after 22 days of acclimatization [13]. It is possible that the adaptations that improve exogenous carbohydrate oxidation with acclimatization develop within only a few days of HA exposure, which would account for the performance enhancing effects observed by Fulco et al. [9] after 3 days of acclimatization. That, combined with the longer duration time trial (greater reliance on carbohydrates) in the Fulco study [9] (720 kJ cycle ergometry time trial), compared to our 2-mile time trial, may explain why they observed improvements in time trial performance and we did not.

Our chronic HA results are in line with what has been previously reported about the influence of carbohydrate supplementation on time trial performance after acclimatization [9, 11]. Although we demonstrated that ability to oxidize exogenous carbohydrates was restored with acclimatization to HA [13], carbohydrate supplementation still did not improve performance. However, exercise performance in both carbohydrate and placebo following 22 days of acclimatization and energy deficit did improve relative to acute HA as a result of the normal adaptive responses that occur with acclimatization. Since muscle glycogen utilization is related to relative exercise intensity (%VO_2peak_) [9, 11, 18], it may be that the exercise intensity during the time trials was not high enough to cause decreases in muscle glycogen that would cause the body to rely on exogenous sources of carbohydrate to complete the exercise task.

The practical implications of our findings must be interpreted in the context of certain experimental limitations. For example, it may be that carbohydrate supplementation did not improve performance due to the potential that such high carbohydrate intakes, particularly at altitude, may have reduced gastric emptying and intestinal carbohydrate absorption [19]. However, those measures were not included in our study. Likewise, our applied performance outcomes may have been strengthened if additional mechanistic measures oxygen transport capacity were included. Our design also does not allow us to completely rule out the potential effects of sustained energy deficit on time trial performance after 22 days of acclimatization. However, including fully fed, energy balance control groups was impractical and outside the scope of our study. We also contend that our findings, which show that carbohydrate supplementation failed to enhance performance following 22 days of acclimatization and concomitant energy deficit, are not only consistent with Fulco et al. [9], but are actually strengthened by our experimental design. More specifically, rather than experimentally enforcing energy balance, we assessed the putative performance enhancing effect of supplemental carbohydrate in response to the real world conditions (i.e., negative energy balance) that lowlanders typically experience during prolonged HA sojourns [10]. Most importantly, considering carbohydrate supplementation did not affect performance, and reliance on endogenous carbohydrate to fuel steady-state exercise appears to be higher during acute HA exposure than it is for absolute intensity-matched exercise at sea level [20], provides practical evidence to recommend that individuals planning to sojourn at HA to complete physically demanding work or exercise should prioritize carbohydrate intake at SL to optimize glycogen stores before ascending to HA.

## Conclusion

We conclude that carbohydrate supplementation during steady-state exercise does not enhance exercise performance in lowlanders acutely exposed to hypoxia or sojourning at HA for 22 days.

## Data Availability

The datasets used and/or analyzed during the current study are available from the corresponding author on reasonable request.

## Abbreviations

CHO: Carbohydrate
HA: High altitude
PLA: Placebo
RPE: Rating of Perceived Exertion
SL: Sea level
SpO_2_: Oxygen saturation
USARIEM: US Army Research Institute of Environmental Medicine
